# miR-302b enhances breast cancer cell sensitivity to cisplatin by regulating E2F1 and the cellular DNA damage response

**DOI:** 10.18632/oncotarget.6381

**Published:** 2015-11-25

**Authors:** Alessandra Cataldo, Douglas G. Cheung, Andrea Balsari, Elda Tagliabue, Vincenzo Coppola, Marilena V. Iorio, Dario Palmieri, Carlo M. Croce

**Affiliations:** ^1^ Department of Molecular Virology, Immunology and Medical Genetics, College of Medicine and Solid Tumor Biology Program, Comprehensive Cancer Center, The Ohio State University, Columbus, OH, USA; ^2^ Department of Biomedical Sciences for Health, University of Milan, Milan, Italy; ^3^ Molecular Targeting Unit, Fondazione IRCCS Istituto Nazionale dei Tumori of Milan, Milan, Italy; ^4^ Start Up Unit, Fondazione IRCCS Istituto Nazionale dei Tumori of Milan, Milan, Italy

**Keywords:** breast cancer, miR-302b, cisplatin, E2F1, ATM, DDR

## Abstract

The identification of the molecular mechanisms involved in the establishment of the resistant phenotype represents a critical need for the development of new strategies to prevent or overcome cancer resistance to anti-neoplastic treatments.

Breast cancer is the leading cause of cancer-related deaths in women, and resistance to chemotherapy negatively affects patient outcomes. Here, we investigated the potential role of miR-302b in the modulation of breast cancer cell resistance to cisplatin.

miR-302b overexpression enhances sensitivity to cisplatin in breast cancer cell lines, reducing cell viability and proliferation in response to the treatment. We also identified E2F1, a master regulator of the G1/S transition, as a direct target gene of miR-302b. E2F1 transcriptionally activates ATM, the main cellular sensor of DNA damage. Through the negative regulation of E2F1, miR-302b indirectly affects ATM expression, abrogating cell-cycle progression upon cisplatin treatment. Moreover miR-302b, impairs the ability of breast cancer cells to repair damaged DNA, enhancing apoptosis activation following cisplatin treatment.

These findings indicate that miR-302b plays a relevant role in breast cancer cell response to cisplatin through the modulation of the E2F1/ATM axis, representing a valid candidate as therapeutic tool to overcome chemotherapy resistance.

## INTRODUCTION

Breast cancer is the leading cause of cancer-related deaths in women [1]. Clinically, this heterogeneous disease is categorized into four major molecular subtypes: Luminal A, Luminal B, HER2 type and triple-negative/basal-like. Triple-negative breast cancer (TNBC) constitutes approximately 15 to 20% of all breast cancer cases, with the worst outcome of all subtypes [2]. For patients affected by TNBC, targeted therapies are not available and chemotherapy has a limited duration of effect in later stages of the disease [3].

Patients usually display a good initial response to cisplatin-based chemotherapy. However, drug resistance is a fundamental problem in breast cancer management, and is responsible for most cases of treatment failure in patients with metastatic cancer [4, 5]. Cisplatin cytotoxicity to normal tissues and cancer cell acquired resistance reduce the clinical efficacy of this drug [6]. However, the molecular mechanisms determining breast cancer resistance to this drug still remain not completely understood.

MicroRNAs (miRNAs) are short (19-22 nucleotide) non-coding RNAs known to alter gene expression at the post-transcriptional level [7, 8]. MicroRNA expression profiling was shown to be associated with tumor development, progression and response to therapy, suggesting their possible use as diagnostic, prognostic and predictive biomarkers [9]. Presumably, miRNAs evolved to allow organisms and cells to effectively deal with cellular stress[10]. Recent studies demonstrated that a single miRNA can impact hundreds of targets [11], and that multiple miRNAs can affect a single target [12], pointing out to the broad implications of miRNAs in the modulation of important cellular processes. Indeed, several experimental and clinical findings have also implicated miRNAs in the response to chemotherapy [13], demonstrating a role for miRNAs in the modulation of genes involved in DNA repair [14, 15].

The miR-302 cluster, which consists of miR-302a, -302a*, -302b, -302b* -302c, -302c*, -302d, -367 and -367*, was first found to be functionally correlated with self-renewal and proliferation properties in the stemness maintenance of embryonic stem cells (ESCs) [16, 17]. Furthermore, tumor-related miRNA studies proved the potential role of miR-302b as tumor-suppressor in different cancer models [18-25]. miR-302b is also reported to be down modulated, compared to normal tissues, in breast cancer [26]. Recently, by microarray analysis, miR-302b was identified as closely associated with the occurrence and development of breast cancer [27]. Moreover, Liang Z. *et al*. demonstrated that miR-302a sensitized radioresistant breast cancer cells to radiation therapy *in vitro* and *in vivo* and reduced the expression of AKT1 and RAD52 [28].

In a previous work, we demonstrated that miR-302b represents a biomarker in human ovarian carcinoma cells able to predict response to cisplatin treatment [29]. In this study we aimed to demonstrate that miR-302b sensitizes breast cell lines to cisplatin treatment by regulating E2F1 expression and cellular DNA Damage Response (DDR) mechanisms.

## RESULTS

### miR-302b sensitizes breast cancer cell lines to cisplatin treatment

Our previous findings demonstrated that high levels of miR-302b represent a predictive biomarker for the response to cisplatin treatment of ovarian cancer cells. Moreover, reduced levels of miR-302b in breast cancer tumors suggest a potential role for this microRNA as a tumor suppressor. Based on these observations, we first aimed to assess the ability of miR-302b to mediate breast cancer sensitivity to cisplatin.

**Figure 1:**
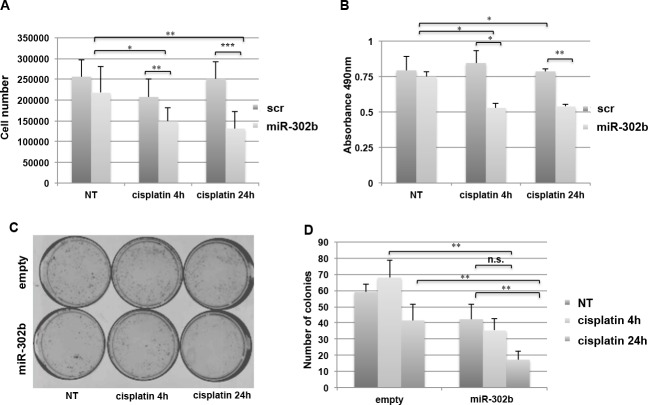
miR-302b enhances cisplatin sensitivity in breast cancer cell lines **A.** MDA-MB-231 cell viability was analyzed after miR-302b precursor or scrambled transfection and after 4 and 24 hours of cisplatin treatment. Cells were stained with Trypan blue and counted. Data are representative of three independent experiments performed at least in triplicate. **B.** MTS assay on MDA-MB-231 cells transfected with miR-302b precursor or scrambled and treated 4 or 24 hours with cisplatin. Absorbance at 490nm (according to manufacturers' instructions) is reported. Data are the average of two independent experiments performed at least in triplicate. **C.** Colony formation assay from MDA-MB-231 cells transfected with a miR-302b expression vector compared to the empty vector control. **D.** Quantitative analysis of the experiment shown in (C). *P*-values were calculated using two-tailed Student's t-test. *=*p* < 0.05; **=*p* < 0.01; ***=*p* < 0.001

To this aim, MDA-MB-231 TNBC cells were transiently transfected with miR-302b precursor or scrambled control (miR-302b and scr). Forty-eight hours following transfection, cells were treated with cisplatin (100μM) or left untreated for 4 or 24 hours. Transfection efficiency was evaluated by RealTime PCR as shown in Supplementary Figure 1A. As shown in Figure 1A, miR-302b transfection enhanced breast cancer cell sensitivity to cisplatin. Similar results were also obtained using different TNBC (BT549) and luminal breast cancer (T47D) cell lines (Supplementary Figure 1B and 1C), indicating that miR-302b modulates cisplatin resistance in different *in vitro* models of breast cancer. MTS assay performed on MDA-MB-231 cells confirmed the inhibitory effects of miR-302b on cell growth following cisplatin treatment (Figure 1B).

To further confirm that miR-302b overexpression enhances cisplatin sensitivity of breast cancer cells, colony assay was also performed. MDA-MB-231 cells were transfected with miR-302b expression vector or with the corresponding empty vector, and treated with cisplatin for 4 and 24 hours. As shown in Figure 1C-1D, a significant reduction in the number of colonies was observed when MDA-MB-231 cells were transfected with miR-302b, compared to empty-transfected cells, following cisplatin treatment.

These results demonstrate that miR-302b sensitizes breast cancer cells to cisplatin treatment.

### miR-302b directly targets E2F1

**Figure 2:**
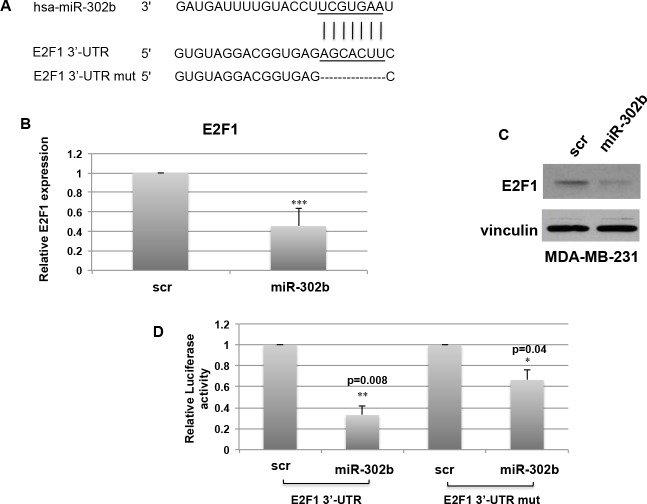
miR-302b directly targets E2F1 **A.** Schematic representation of miR-302b targeting site on wild type and mutant E2F1-3′UTR. **B.**-**C.** Real Time PCR **B.** and Western Blot **C.** evaluation of E2F1 expression levels on MDA-MB-231 cells transfected with miR-302b precursor or a scrambled oligonucleotide after 48h. **D.** Luciferase activity of E2F1-3′UTR and E2F1-3′UTR mutated plasmids transfected in MDA-MB-231 cells in the presence of miR-302b precursor or scrambled negative control. Data are the average of two independent experiments +S.D. calculated on 3 different replicates. *P*-values were calculated using two-tailed Student's *t*-test. *=*p* < 0.05; **=*p* < 0.01; ***=*p* < 0.001

To identify the mechanisms by which miR-302b sensitizes breast cancer cells to cisplatin treatment, we performed an *in silico* analysis of the potential target genes of this microRNA. TargetScan algorithm (www.targetscan.org) identified E2 transcriptor factor 1 (E2F1) as predicted target gene for miR-302b (Figure 2A). The E2F family of transcription factors plays a crucial role in the control of cell proliferation, regulating the expression of many genes required for entry and progression through the S phase of the cell cycle [30]. Moreover, E2F1 protein is stabilized and its levels are increased in response to DNA damage [31-33].

As shown in Figure 2B-2C, miR-302b overexpression in MDA-MB-231 cells resulted in the decrease of E2F1 expression both at the mRNA (Figure 2B) and protein (Figure 2C) levels.

To demonstrate the direct targeting of E2F1 3′-UTR by miR-302b, a luciferase reporter assay was performed. To this aim, the 3′-UTR region of E2F1 including the predicted binding site for miR-302b was cloned downstream the luciferase reporter gene. MDA-MB-231 cells were co-transfected with miR-302b precursor or scrambled negative control and the reporter vector. As shown in Figure 2D, a significant decrease in luciferase activity was observed in miR-302b transfected cells as compared to scrambled transfected cells. Of note, deletion of the miR-302b binding site on the E2F1 3′-UTR reporter vector (Figure 2A) partially rescued the inhibitory effect of miR-302b on luciferase expression (Figure 2D).

Taken together, these findings indicate that E2F1 is a new target of miR-302b.

### miR-302b regulates ATM through E2F1

**Figure 3:**
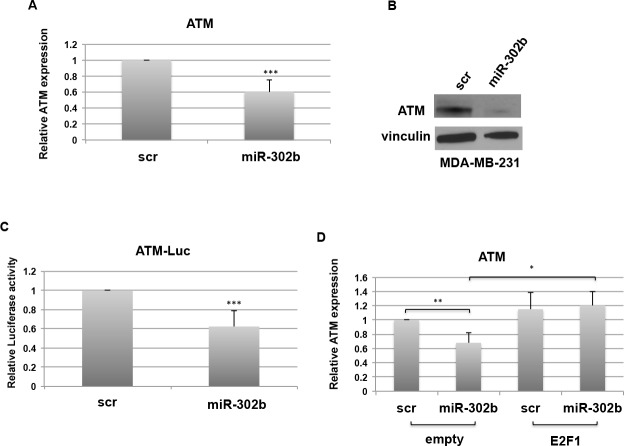
miR-302b indirectly targets ATM **A.**-**B.** MDA-MB-231 cells were transfected with miR-302b precursor or scrambled control and RNA and proteins were collected after 48h. ATM mRNA levels were analyzed by RT-qPCR **A.** and protein expression was evaluated by Western Blot **B. C.** Relative Luciferase activity in MDA-MB-231 cells for ATM human promoter co-transfected with miR-302b precursor or scrambled for 48h. **D.** ATM expression after miR-302b precursor or scrambled transfection in the presence of E2F1 expression vector or its empty control in MDA-MB-231. *=*p* < 0.05; **=*p* < 0.01; ***=*p* < 0.001

ATM (Ataxia-Telangiectasia Mutated) is one of the most important serine/theronine kinase that transduces DNA damage signals to downstream mediators involved in the cellular DDR [34, 35]. ATM is primarily activated in response to DNA Double Strand Breaks (DSBs) due to cisplatin treatment, mediating cell cycle arrest, repair of damaged DNA and cellular apoptosis [14]. It was previously demonstrated that E2F1 activates ATM promoter activity, resulting in increased ATM mRNA and protein levels [36]. Since miR-302b negatively regulates E2F1, we hypothesized that up-regulation of this microRNA could result in reduced transcriptional activation of ATM promoter. Accordingly, we observed a significant decrease of both ATM mRNA and protein levels when miR-302b was expressed in MDA-MB-231 cells (Figure 3A and 3B). However, *in silico* analysis of ATM 3′UTR did not identify potential binding sites for miR-302b (data not shown). Then, we hypothesized that miR-302b could indirectly affect ATM expression through E2F1. To validate this hypothesis, we performed a luciferase assay by transfecting MDA-MB-231 cells with a reporter plasmid containing the luciferase gene under the transcriptional control of human ATM gene [36] in the presence of miR-302b precursor or a scrambled negative control. Figure 3C shows that the transcriptional activity of ATM promoter was significantly reduced by miR-302b transfection compared to scrambled transfected cells (Figure 3C). To confirm that miR-302b-mediated regulation of ATM gene expression was dependent on E2F1 transcriptional activity, MDA-MB-231 cells were transfected with miR-302b precursor or a scrambled negative control following transfection of E2F1 expression vector (not including its 3′-UTR) or its empty negative control (Supplementary Figure 2A-2B). Real Time analysis confirmed the down regulation of ATM expression following miR-302b transfection. Conversely, exogenous expression of E2F1 rescued the expression of ATM also in the presence of miR-302b.

Finally, we demonstrated that miR-302b negatively regulates E2F1 and, indirectly, ATM, using two different cellular models of breast cancer, BT-549 (TNBC) and T47D (luminal breast cancer). As shown in Supplementary Figure 2C-2E, exogenous expression of miR-302b resulted in downregulation of E2F1 and ATM also in these systems.

Taken together, these data indicate that miR-302b, by targeting E2F1, negatively affects ATM expression levels in breast cancer cells of different origin.

### miR-302b affects cell cycle progression after cisplatin treatment

**Figure 4:**
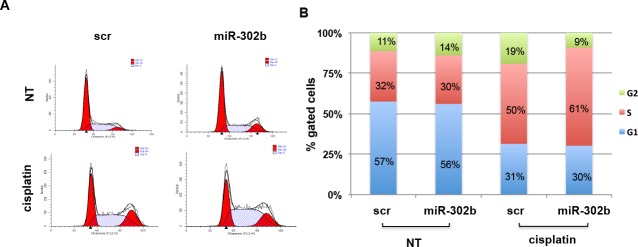
miR-302b affects cell cycle progression after cisplatin treatment in MDA-MB-231 **A.** Cell cycle analysis of MDA-MB-231 transfected with miR-302b precursor or scrambled and treated with cisplatin. Cells were fixed and stained with propidium iodide and analyzed by flow cytometry. Data obtained were analyzed using ModFit software. Cells in G1 and in G2 phase of cell cycle are reported in red, cells in S phase are indicated with white and blue bars. Flow cytometry plots shown are representative of three independent experiments. **B.** Graphic representation of cell distribution in G1, S, or G2 phase (blue, red and green, respectively) of the experiment shown in **A.**. Percentages of cells in each phase of the cell cycle are the average of three independent experiments.

Since E2F1 regulates cell proliferation by modulating S-phase entry and progression, we decided to investigate the potential role of miR-302b in cell cycle control in response to cisplatin treatment. MDA-MB-231 (Figure 4) and BT549 (Supplementary Figure 3) breast cancer cells were transiently transfected with miR-302b precursor or scrambled negative control, treated for 4h with cisplatin, collected at 24h from the treatment and analyzed by flow-cytometry following propidium-iodide staining. No significant alteration of cell cycle distribution was observed between untreated miR-302b-transfected or scrambled-transfected MDA-MB-231 cells. Conversely, following cisplatin treatment, miR-302b-transfected cells displayed a higher accumulation in the S phase of the cell cycle higher compared to negative control-transfected cells (61% vs 50%). Similar results were obtained using BT549 cells following miR-302b transfection and cisplatin treatment (Supplementary Figure 3). These results indicate that overexpression of miR-302b inhibits cell-cycle transition through S-phase following cisplatin treatment of breast cancer cells.

### miR-302b overexpression impairs Homologus Recombination and Non-Homologus End Joining DNA repair mechanisms

**Figure 5:**
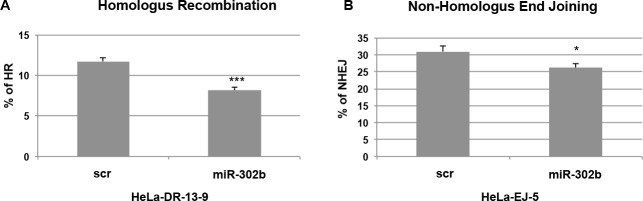
miR-302b affects DNA repair mechanisms **A.** Homologous recombination (HR) and **B.** Non-Homologus End Joining (NHEJ) assays using HeLa-DR-13-9 and HeLa-EJ-5 cells (respectively) transfected with miR-302b precursor or scrambled control (scr). After generating a double-strand DNA break by expressing the *I-SceI* endonuclease, functional HR and NHEJ results in the conversion of cells to positive for GFP. The percentage of GFP-positive cells was determined by flow cytometry. Reported data are representative of two experiments performed in triplicate and mean + SD is reported. Statistical significance was analyzed by the unpaired Student's *t*-test. *=*p* < 0.05; **=*p* < 0.01; ***=*p* < 0.001

Our data so far demonstrated that miR-302b overexpression in breast cancer cell lines leads to sensitization to cisplatin treatment, by targeting E2F1 and ATM. Since ATM plays a key role in the molecular mechanisms of DNA repair, we also investigated whether miR-302b overexpression could result in the impairment of these mechanisms, cycle checkpoint activation and, eventually, apoptosis or senescence [37]. Following DNA DSBs, the two major pathways involved in DNA repair are Homologous Recombination (HR) and Non-Homologous End Joining (NHEJ) [38]. We evaluated the effects of miR-302b overexpression on both DDR mechanisms using two established assays based on HeLa derived cells (HeLa-DR 13-9 and HeLa-EJ-5 for HR and NHEJ, respectively) with the specific recombination substrate DNA integrated in the genome to study each DSB repair pathway [39, 40]. In both systems, if HR-mediated or NHEJ DNA-repair occurs, the recombination generates an active GFP allele that can be revealed by detection and quantification of green-fluorescent cells. We transfected HeLa-DR13-9 and HeLa-EJ5 cells with either a miR-302b precursor or scrambled control and, two days later, with plasmid encoding for the *I-SceI* endonuclease to induce DNA damage. The percentage of GFP-positive cells (reflecting the efficiency of HR-mediated and NHEJ DNA-repair) was then assessed by flow cytometry at 72 h after the second transfection. As shown in Figure 5A and 5B, miR-302b expression resulted in about 30% and 15% reduction of GFP-positive cells. RNA was extracted from HeLa-DR13-9 cells to verify miR-302b transfection and down-regulation of E2F1 and ATM mRNA levels (Supplementary Figure 4). All together these results indicate that miR-302b improve sensitivity to chemotherapy in breast cancer cell lines negatively affecting DNA-repair.

### miR-302b overexpression and cisplatin treatment induce apoptosis

**Figure 6:**
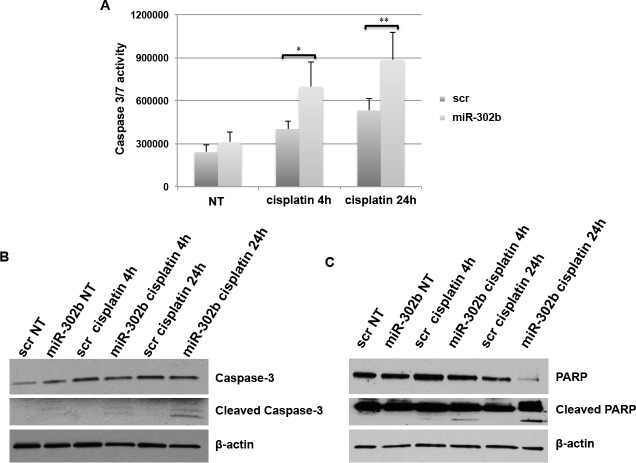
miR-302b effects on apoptosis **A.** Caspase 3/7 activation assay following transient transfection of MDA-MB-231 cells with miR-302b precuror or scrambled control and upon cisplatin treatment for 4 and 24 hours. Data are the average of three independent experiments. P-values were calculated using two-tailed Student's *t*-test. *=*p* < 0.05; **=*p* < 0.01; ***=*p* < 0.001. **B.**-**C.** Caspase-3 **B.** and PARP **C.** protein expression evaluated by Western Blot after miR-302b or scrambled transfection and cisplatin treatment for 4 and 24 hours in MDA-MB-231 cells.

We have demonstrated that miR-302b sensitizes breast cancer cell lines to cisplatin treatment by targeting directly E2F1 and indirectly ATM, reducing cell growth. To evaluate whether miR-302b expression could also activate apoptosis in response to cisplatin treatment, we performed Caspase 3/7 activation following transient transfection of miR-302b in MDA-MB-231 cells. As shown in Figure 6A, significant Caspase activation induction was observed at 4 and 24 hours upon cisplatin treatment (Figure 6A). Similarly, miR-302b overexpression resulted in enhanced Caspase 3/7 activation also in BT549 cells (Supplementary Figure 5).

Accordingly, as shown in Figure 6B and 6C, western blot analysis of MDA-MB-231 transiently transfected with miR-302b and treated with cisplatin 24h showed a significant PARP degradation and Caspase-3 activation compared to scrambled negative control transfected cells.

These results demonstrated that miR-302b overexpression enhances cisplatin sensibility of breast cancer cells, by inducing apoptosis.

## DISCUSSION

Breast cancer patients usually have a good initial response to cisplatin-based chemotherapy but the insurgence of drug resistance represents one of the major causes of treatment failure, especially for patients with metastatic disease [4, 5].

A growing body of evidence demonstrated a correlation between miRNAs expression and tumor chemo- and radiosensitivity [28, 41, 42], further supporting the strong clinical relevance of microRNAs.

In a previous study, we demonstrated that miR-302b acts as chemo-sensitizer in human ovarian carcinoma cell lines by targeting HDAC4, and may represent a biomarker able to predict response to cisplatin [29].

The aim of this study was to evaluate the role of miR-302b in the modulation of breast cancer cell sensitivity to cisplatin, also investigating the molecular mechanisms affected by this microRNA and involved in cancer cell resistance to these treatments.

Here, we first analyzed cisplatin sensitivity of three different breast cancer cell lines, MDA-MB-231 and BT549 (triple negative) and T47D (luminal), upon miR-302b overexpression. Our results indicate that miR-302b overexpression induces a significant decrease of cell viability and proliferation following cisplatin treatment in different breast cancer cell lines, suggesting that miR-302b could play an important role in mediating cisplatin sensitivity of both TNBC and luminal breast cancer cells.

*In silico* analysis allowed us to the identify E2F1, a master regulator of the G1/S transition, as a new potential miR-302b target. Our experimental data demonstrated that miR-302b negatively modulates E2F1 expression at both mRNA and protein levels. Luciferase assays confirmed the direct targeting of E2F1 3′-UTR by miR-302b. However, mutation of E2F1 3′-UTR sequence in the potential miR-302b target site only partially abrogated miR-302b control of E2F1 expression, suggesting that other non-canonical binding sites for this microRNA could be present.

Accordingly with the role of E2F1 in the control of cell-cycle progression, we confirmed that down-regulation of this protein upon miR-302b overexpression results in a slower transition through the S-phase of the cell cycle, following cisplatin treatment. Of note, major alterations of the cell cycle progression were not observed in untreated cells, suggesting that other factors, such as the other members of the E2F family, might compensate for the absence of E2F1 following miR-302b overexpression. However, following cisplatin treatment, the stronger accumulation in the G1/S phase of the cell cycle mediated by miR-302b suggests the presence of specific molecular pathways relying on E2F1.

It has been widely demonstrated that E2F1 elevates ATM promoter activity and enhances ATM mRNA and protein levels [36]. This protein activates a signaling cascade that leads to the immediate triggering of DNA-repair mechanisms, [34, 43] and it is primarily activated in response to DSBs due to cisplatin treatment [14]. Usually, cells activate multiple DNA repair mechanisms to remove cisplatin-damaged DNA. These DNA repair systems are detrimental to the cytotoxic efficacy of the drug. Indeed, increased DNA repair capacity of cancer cells is a key mechanism involved in cisplatin resistance [44]. Since our data demonstrated that miR-302b directly targets E2F1, we also analyzed ATM expression. Interestingly we found that miR-302b negatively regulates ATM expression indirectly, at least in part through the direct down-regulation of E2F1 expression. In the presence of higher levels of miR-302b, reduced levels of E2F1 result in the partial abrogation of ATM activation, affecting cancer cell ability to repair DNA and proliferate. Accordingly, here we show that exogenous expression of miR-302b negatively affects the ability of breast cancer cells to repair damaged DNA by NHEJ and HR, which suggests that miR-302b correspond to cellular alteration following DNA-damaging agents treatment.

Consequently, down-regulation of ATM-dependent mechanisms of DDR leads to enhanced cell death following cisplatin treatment. In fact, our results show apoptosis activation following miR-302b overexpression and cisplatin treatment, as indicated by Caspase-3 and PARP cleavage.

The advantage of miRNAs is their ability to affect multiple targets with a single hit, thus resulting a whole network of interacting molecules. However, we found that miR-302b, through the negative regulation of E2F1, targets ATM and we could confirm that miR-302b replacement therapy might enhance sensitivity to chemotherapy in breast cancer cells, targeting genes involved in mechanisms such as cell cycle and DDR frequently altered in cancer.

In conclusion our study demonstrates that miR-302b overexpression increases sensitivity of breast cancer cell lines to cisplatin by targeting E2F1 and ATM, further compromising the control of cell-cycle progression and DDR mechanisms.

These findings indicate that miR-302b might represent a biomarker to predict response to cisplatin treatment in breast cancer patients, supporting this microRNA as potential therapeutic tool to overcome chemotherapy resistance.

## MATERIALS AND METHODS

### Cell lines, transfections and treatments

All the cell lines used were purchased from the American Type Culture Collection (ATCC). MDA-MB-231, BT549 and T47D cells were grown in Roswell Park Memorial Institute 1640 (RPMI) medium, containing 10% heat-inactivated fetal bovine serum (FBS), 2 mM l-glutamine and 100 U/mL penicillin-streptomycin. MDA-MB-231, BT-549 and T47D were treated for 4 and 24 hours with cisplatin 100 μM (Sigma).

For transfection experiments, cells were seeded in 6-well plates at 2×10^5^/well or in 96-well at 2×10^3^/well and transfected with 100nM of hsa-miR-302b-3p Pre-miR^TM^ miRNA Precursor (Ambion) or Pre-miR^TM^ Negative Control #1 (Ambion) or different amounts (5-200ng) of indicated plasmid DNAs. All transfections were carried out with Lipofectamine 2000 (Invitrogen, Carlsbad, CA) according to the manufacturer's instructions.

### Quantitative real-time PCR for miRNA and mRNA quantification

Total RNA was extracted using TRIzol (Invitrogen) according to manufacturer's instructions. Quantitative real-time PCR (qRT-PCR) were performed using the TaqMan Fast-PCR kit (Applied Biosystems) according to the manufacturer's instructions, using the appropriate TaqMan probes for miRNA and gene quantification, followed by detection with the 7900HT Sequence Detection System (Applied Biosystems). All reactions were performed in triplicate. Simultaneous quantification of RNU44 was used as reference for miRNA quantification. Simultaneous quantification of GAPDH mRNAs was used as reference for gene quantification. The comparative cycle threshold (Ct) method for relative quantification of gene and miRNA expression (User Bulletin #2; Applied Biosystems) was used to determine miRNA and gene expression levels.

### Cell viability

MDA-MB-231, BT549 and T47D cells seeded in 6 wells at a density of 2×10^5^ cells/well were transfected with 100nM hsa-miR-302b-3p Pre-miR^TM^ miRNA Precursor (Ambion) or Pre-miR^TM^ Negative Control #1 (Ambion). After 48h of culture, cells were treated or not with cisplatin for 4 and 24 hours. Cells were counted using Trypan Blue Stain 0.4% (GIBCO) at 24h following the treatment. Data are representative of at least two independent experiments performed in triplicate and mean + SD is reported. Statistical significance was analyzed by the unpaired Student t-test.

### MTS assay

MDA-MB-231 cells seeded in 96 wells at a density of 2×10^3^ cells/well were transfected with 100nM hsa-miR-302b-3p Pre-miR^TM^ miRNA Precursor (Ambion) or Pre-miR^TM^ Negative Control #1 (Ambion). After 48h of culture, cells were treated or not with cisplatin for 4 and 24 hours. Cells were then incubated with MTS solution (Promega) according to manufacturer's instructions. Cell growth was assessed based on optical density (OD) at 490nm using a spectramax 340 microtiter microplate reader (Molecular Devices). The mean ± S.D. of two independent experiments performed in triplicate is reported. Statistical significance was analyzed by the unpaired Student t-test.

### Colony forming assay

MDA-MB-231 cells were plated in 100-mm dishes and transfected with 500ng of miR-302b expression plasmid or empty control (OriGene). After 48 h, Geneticin (Invitrogen) was added at the final concentration of 500 μg/mL. Three weeks after the onset of drug selection, colonies were stained with crystal violet acquired using the QuantityOne software (Biorad) and counted. Data are representative of one experiment performed in triplicate and mean + SD is reported. Statistical significance was analyzed by the unpaired Student t-test.

### Plasmid construction

The E2F1 3′ untranslated region (UTR) reporter construct, including the binding site for miR-302b and cloned into the pGL3 control vector (Promega, Madison, WI) downstream of the luciferase gene was previously described [45]. Mutations of the miR-302b binding site in the E2F1 3′-UTR were introduced using the Quick-Change Lightning Site-Directed Mutagenesis kit (Agilent Technologies, Santa Clara, CA), according to the manufacturer's instructions.

Primers for plasmid construction and mutagenesis were:

E2F1 Fw: 5′-TCTAGACTTGGAGGGACCAGGGTTTC-3′
E2F1 Rev: 5′-TCTAGAAAAGCAGGAGGGAACAGAGC-3′
E2F1 Mut Fw 5′-gcgtgtaggacggtgagctgtcttaaaggtttttt-3′ E2F1 Mut Rev 5′-aaaaaacctttaagacagctcaccgtcctacacgc-3′

The luciferase reporter plasmid ATM-Luc containing the human ATM promoter was kindly provided by Dr. Ginsberg (Department of molecular Cell Biology, The Weizmann Institute of Science, Rehovot 76100, Israel). The E2F1 expression vector was purchased from Addgene (Plasmid #21667) [46].

### Luciferase assays for target and promoter identification

Luciferase reporter vectors (see above) and 10 ng of the pRL-SV40 (Renilla) control vector (Promega), and 100 nM miR-302b or scrambled sequence miRNA control (Ambion Inc, Austin, TX) were co-transfected into MDA-MB-231 cells in 12-well plates. Firefly luciferase activity was measured with the Dual Luciferase Assay Kit (Promega) 24h after transfection and normalized for the Renilla luciferase reference plasmid. Reporter assays were carried out in at least in quadruplicate and the mean (representative of at least two independent experiments) ± S.D. was reported. Statistical significance was analyzed by the unpaired Student t-test.

### Western blot analyses

Protein extraction and western blots were performed as previously described [47]. Protein concentration was detected with Bradford method. The antibodies used were: anti-E2F1 (sc-251, Santa Cruz Inc), anti-ATM (sc-23921, Santa Cruz Inc), anti-PARP (9532, Cell Signaling), anti-Caspase-3 (9662, Cell Signaling) anti-β actin (sc-47778, Santa Cruz Inc), anti-vinculin (sc-73614, Santa Cruz Inc).

### Apoptosis assay

Apoptosis was quantified using Caspase-Glo 3/7 assay (Promega) according to the manufacturer's instructions on a Bio-Tek Synergy HT multi detection microplate reader. The assay was performed three times in triplicate and the mean + S.D. was reported. Statistical significance was analyzed by the unpaired Student t-test.

### FACS analysis

Cells were trypsinized, washed in PBS and fixed in 70% ethanol for 2h at −20°. After fixation, cells were washed in PBS and centrifuged for 5 min at 1200 rpm. Cells were resuspended in PBS containing 10mg/ml propidium iodide (Roche) and incubated for 20 min at 37°C. Cells were analyzed using FACS-Calibur flow cytometer and the results were further analyzed with the ModFit software, v3.2 (Verity Software House). The assay was performed three times in triplicate and the mean + S.D. was reported. Statistical significance was analyzed by the unpaired Student t-test.

### DNA repair assays

HR (Homologous Recombination) and NHEJ (Non-Homologous End Joining) assays were performed as previously described [39, 40]. Briefly, HeLA-DR13-9 or HeLa-EJ-5 cells were transfected with 30pmol of hsa-miR-302b-3p Pre-miR^TM^ miRNA Precursor (Ambion) or Pre-miR^TM^ Negative Control #1 (Ambion). On day 2, cells were transferred to 35 mm dishes. On day 3, cells were transfected with 50 pmol of hsa-miR-302b-3p Pre-miR^TM^ miRNA Precursor (Ambion) or Pre-miR^TM^ Negative Control #1 (Ambion) along with *I-SceI* expression vector to induce DNA DSB. On day 6, cells were harvested and GFP positive cells were counted using a FACS Calibur flow cytometer (Becton-Dickinson). For each experimental point, at least 10,000 cells were analyzed. Data are representative of two independent experiments performed in triplicate and mean + SD is reported.

## SUPPLEMENTARY INFORMATION FIGURES



## Abbreviations

E2F1: E2 transcriptor factor 1
ATM: Ataxia-Telangiectasia Mutated
DDR: DNA Damage Response
HR: Homologus Recombination
NHEJ: Non-Homologus End Joining
DSB: Double Strand Breaks
