# Supplementary material for: miR-302b enhances breast cancer cell sensitivity to cisplatin by regulating E2F1 and the cellular DNA damage response
Source: Oncotarget. 2015 Nov 25;7(1):786–97. doi: 10.18632/oncotarget.6381 (PMC4808033; doi:10.18632/oncotarget.6381)
Supplement: Supplementary file 1 [file oncotarget-07-0786-s001.pdf]

miR-302b enhances breast cancer cell sensitivity to cisplatin by regulating E2F1 and the cellular DNA damage response

Supplementary Information

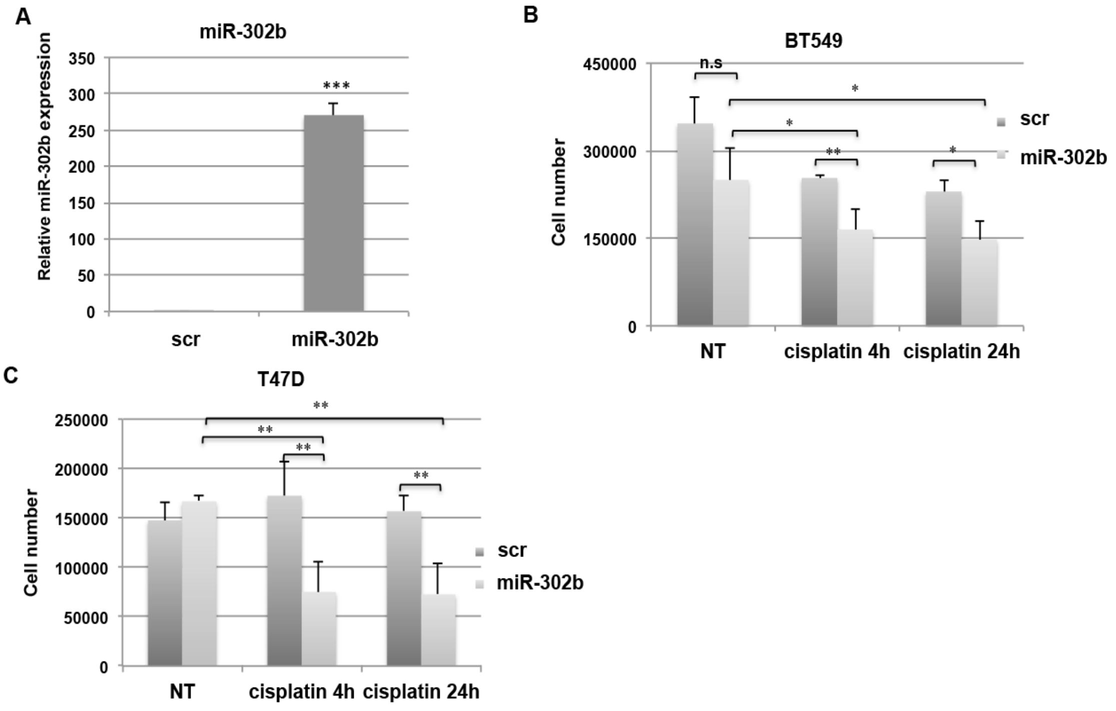

Supplementary Figure 1. miR-302b modulates breast cancer cell resistance to cisplatin. (A) miR-302b expression was assessed by RT-qPCR in MDA-MD-231. (B-C) BT549 and T47D cell viability was analyzed after miR-302b precursor or scrambled transfection and after 4 and 24 hours of cisplatin treatment. Cells were stained with Trypan blue and counted. Data are representative of one experiment performed in quadruplicate. Statistical significance was analyzed by the unpaired Student's t-test. *=p<0.05; **=p<0.01; ***=p<0.001

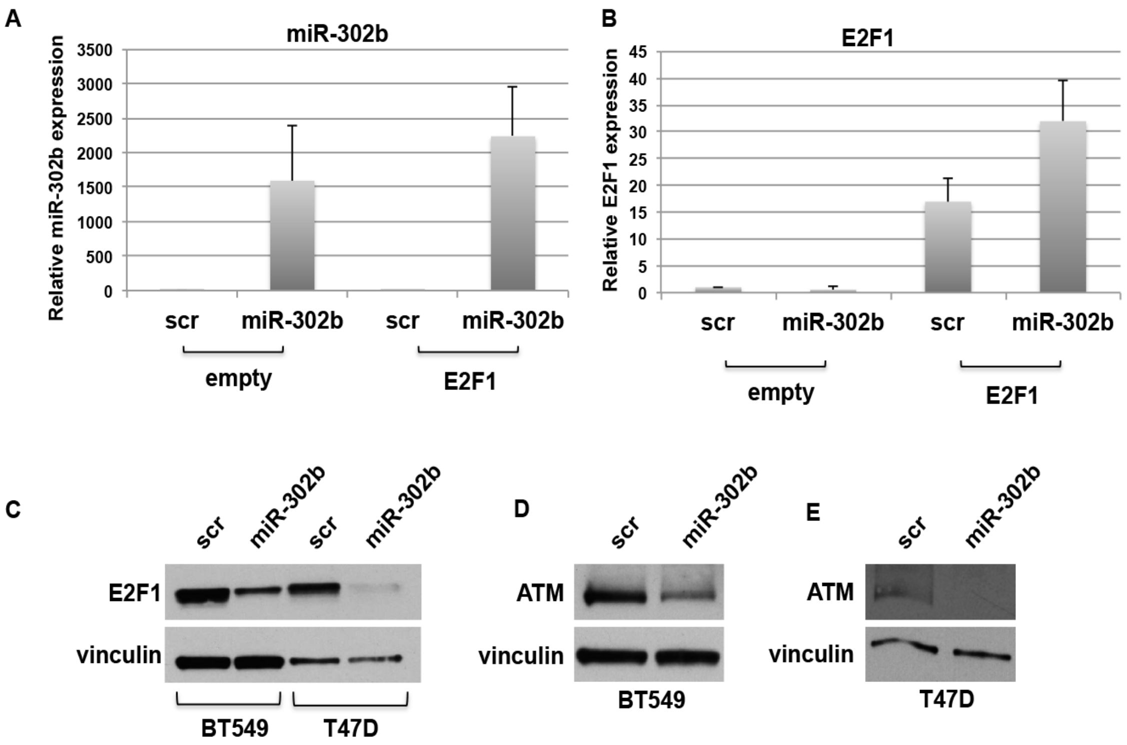

Supplementary Figure 2. miR-302b regulates cellular E2F1 and ATM levels (A-B) miR-302b (A) and E2F1 (B) expression after miR-302b or scrambled transfection in the presence of E2F1 expression vector or its empty control in MDA-MB-231, evaluated by Real Time PCR. (C-E) Analysis of E2F1 and ATM expression levels by Western blot analysis in BT549 and T47D cells following exogenous expression of miR-302b. Vinculin was used as loading control.

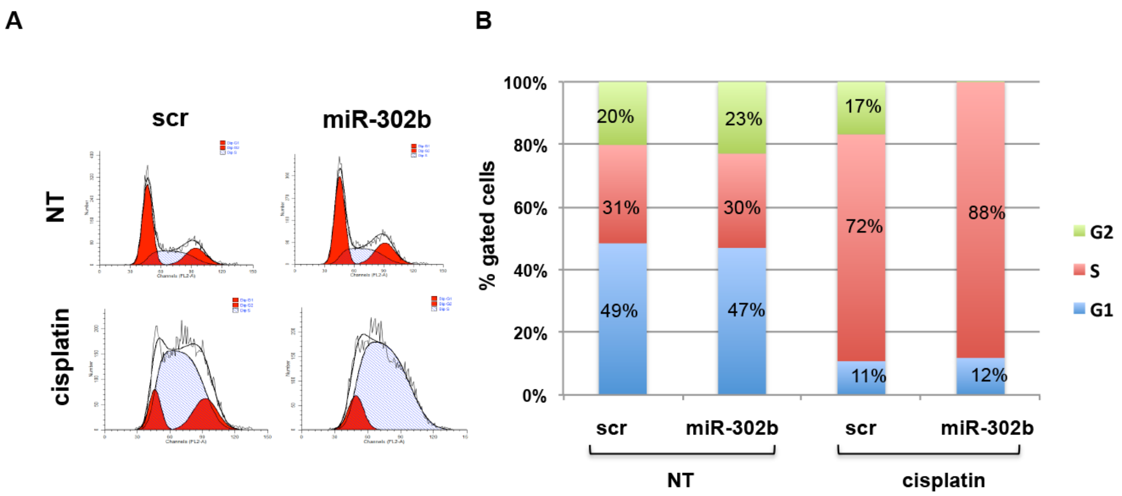

Supplementary Figure 3. miR-302b affects cell cycle progression after cisplatin treatment in BT549. (A) Cell cycle analysis of BT549 transfected with miR-302b precursor or scrambled and treated with cisplatin. Cells were fixed and stained with propidium iodide and analyzed by flow cytometry. Data obtained were analyzed using ModFit software. Cells in G1 and in G2 phase of cell cycle are reported in red, cells in S phase are indicated with white and blue bars. (B) Graphic representation of cell distribution in G1, S, or G2 phase (blue, red and green, respectively) of the experiment shown in (A).

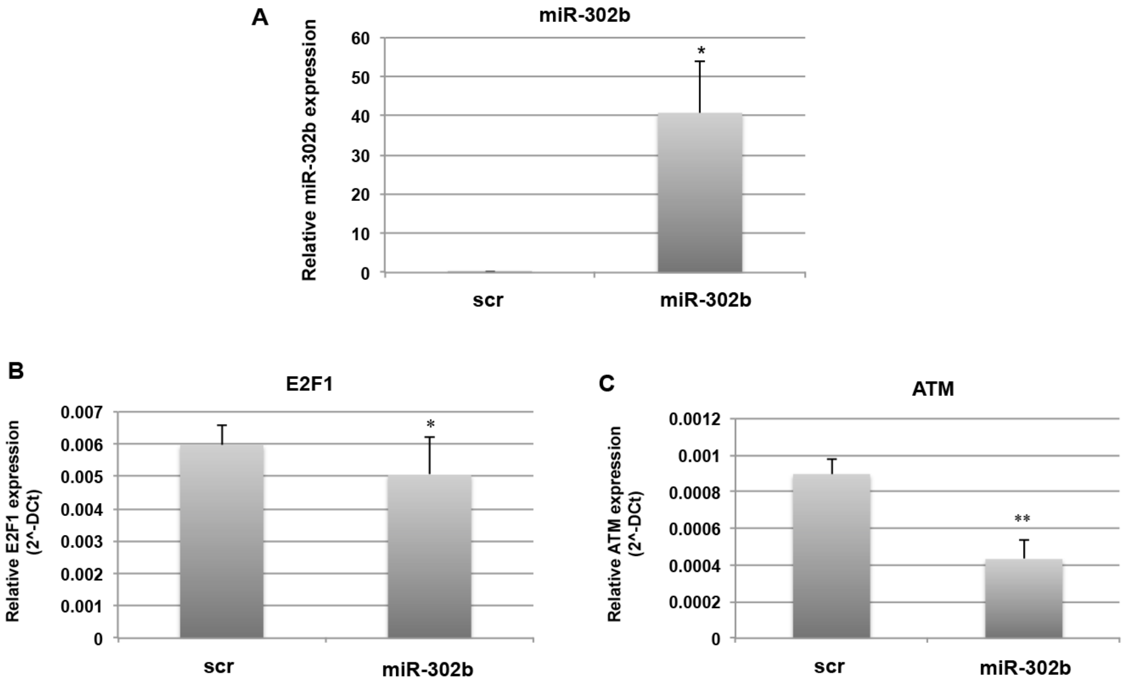

Supplementary Figure 4. miR-302b regulates NHEJ- and HR-mediated DNA repair(A-C) miR-302b (A), E2F1 (B) and ATM (C) expression was assessed by RT-qPCR on HeLa-DR-13-9 and HeLa-EJ-5 cells transfected with miR-302b precursor (HeLa miR-302b) or with scrambled control (HeLa scr). Statistical significance was analyzed by the unpaired Student's t-test. *=p<0.05; **=p<0.01; ***=p<0.001

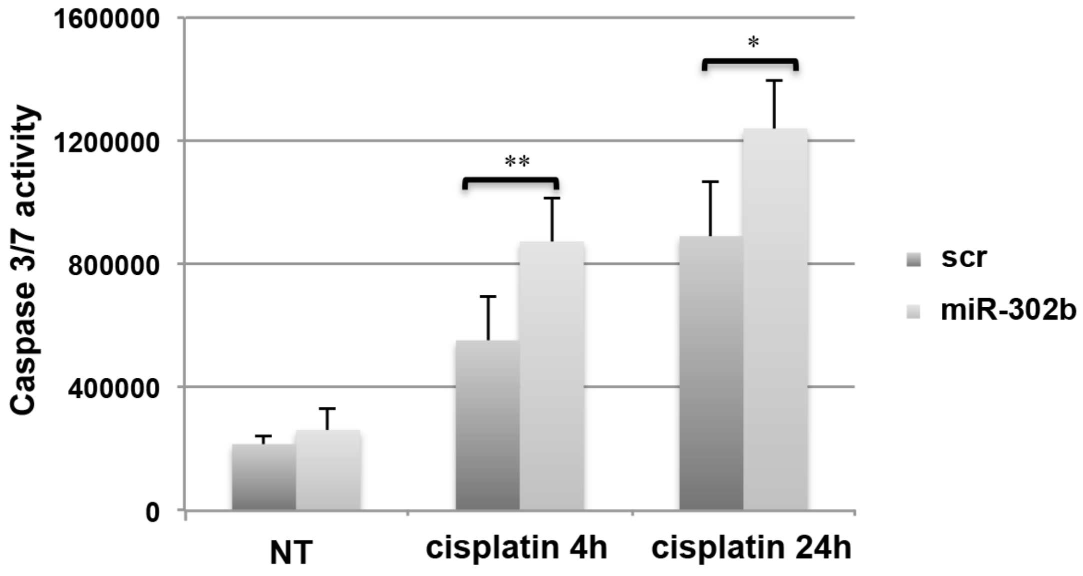

Supplementary Figure 5. **miR-302b induces apoptosis in breast cancer cells following cisplatin treatment.** Caspase 3/7 activation following transient transfection of BT549 cells with miR-302b precursor or scrambled control and upon cisplatin treatment for 4 and 24 hours. Data are representative of two independent experiments performed in triplicate. P-values were calculated using two-tailed Student's t-test. *=p<0.05; **=p<0.01; ***=p<0.001
